# Selective Mesh Augmentation to Prevent Incisional Hernias in Open Colorectal Surgery Is Safe and Cost-Effective

**DOI:** 10.3389/fsurg.2018.00008

**Published:** 2018-02-16

**Authors:** Núria Argudo, Miguel Pera, Manuel López-Cano, Lourdes Hernández, Juan José Sancho, Luis Grande, José Antonio Pereira

**Affiliations:** ^1^Department of General and Digestive Surgery, Parc de Salut Mar, Barcelona, Spain; ^2^Department de Cirurgia, Universitat Autònoma de Barcelona, Barcelona, Spain; ^3^Department de Ciències Experimentals i de la Salut, Universitat Pompeu Fabra, Barcelona, Spain; ^4^Department of General Surgery, Hospital Universitari Vall d’Hebrón, Barcelona, Spain

**Keywords:** incisional hernia, colorectal surgery, prophylactic mesh, mesh augmentation, laparotomy

One of the most common complications in abdominal surgery is the incisional hernia (IH), especially when following a midline laparotomy (1). This condition demands a considerable amount of health-care resources, as it can easily require a new surgery, due to IH complication or to alleviate symptomatology, which deeply affects the patients’ quality of life (2). Despite the advances in surgical techniques and materials, the prevalence of IH remains high, although figures in published studies show great variation, reporting rates of IH between 3 and 20% (3, 4). In high-risk patients, however, the incidence rate can be as high as 40% (1, 5).

In order to avoid the complications resulting from IH, many attempts have been made in the field of IH prevention (4). Initially, most studies focused their strategy in optimizing laparotomy closure (6, 7), with variations regarding suture material and technical advances like the use of retention sutures (8), but they have all proven insufficient. In recent years, a new approach has been introduced to prevent IH, the use of prophylactic synthetic meshes. To date, there are only a few widely accepted indications for a prophylactic mesh, all reserved for special subgroups such as aortic aneurysm surgery, bariatric surgery, or stoma placement (9–11). However, more recent studies suggest that prophylactic mesh augmentation in the closure of midline laparotomy could be a useful alternative to prevent IH in high-risk patients without adding significant morbidity (12, 13).

There are many risk factors identified for IH, such as old age, smoking, previous abdominal surgery, male gender, obesity, and comorbidities such as malnutrition, abdominal aortic aneurysm, chronic renal failure, diabetes, chronic obstructive pulmonary disease (COPD), and immunosuppression (1, 14, 15). Higher BMI is one of the factors strongly associated with IH (16–18).

In a retrospective study conducted in our institution, we reported an incidence rate of IH as high as 39.9% in a series of 338 patients undergoing colorectal resection, identifying two groups of patients with higher risk: obese patients and nonobese patients with the combination of several secondary risk factors for IH (1). These results showed that it was mandatory to come up with improvement measures to secure better outcomes for our patients.

We designed an algorithm, based on this previous study, to help surgeons decide which patients should receive a prophylactic mesh (19). All patients undergoing midline laparotomy for colorectal cancer resection with BMI greater than 29 [the median BMI of patients with IH in our previous study (1)] and those with BMI lower than 29 but with two or more risk factors for IH were considered for mesh augmentation. The rest were assigned to a regular closure.

The following variables were considered as risk factors for IH: BMI (kg/m^2^), smoking, serum creatinine, hemoglobin, serum albumin, COPD, diabetes mellitus, immunosuppression with steroids or previous radiotherapy/chemotherapy, and previous surgery through midline laparotomy.

After implementation of the protocol, we assessed the utility and cost-effectiveness of the algorithm for mesh augmentation.

A prospective cohort study was conducted including all patients undergoing a midline laparotomy for colorectal cancer resection between January 2011 and June 2015.

All the surgeons in the team were trained in the technique of mesh augmentation. The *linea alba* was closed by continuous long-term absorbable suture using polydioxanone n.1 thread (PDS^®^ Ethicon, NJ, USA), following the recommended techniques about suture length, distance between stitches and amount of tissue (6, 7). In the cases with mesh augmentation, it was placed “onlay” after subcutaneous dissection of 3 cm in both sides of the incision. We used a low-weight, wide pore, partially absorbable mesh (Ultrapro^®^, Ethicon, NJ, USA) fixed with a double crown of fascia staples (DFS^®^ Autosuture, Covidien, MA, USA). Closed suction subcutaneous drains were placed in all patients with mesh.

Postoperative complications were recorded according to the Clavien–Dindo classification (20), paying particular attention to wound complications such as evisceration, seroma, and superficial or deep surgical site infection.

Total hospital cost and resource expenses during index laparotomy and subsequent related hospital admissions or IH repairs were obtained. The sample was divided into two groups depending on the application of the algorithm in the initial operation.

The diagnostic criteria for IH were as follows: clinical diagnosis made by a trained surgeon, surgery for IH during the follow-up period, and/or IH detected by the CT-scan routinely performed during the follow-up period.

During the study period, 226 patients, mean age 77 (11) years, 61% male, were included in the analysis. The mean follow-up was 31.5 months (12–60). About 160 patients were treated following the algorithm at a mean cost of 10.057€ (4.339). In 66 patients, the algorithm was not followed and the cost was 10.921€ (4.758). The incidence rate of IH dropped from 43.9 to 10% when the algorithm was followed (*P* = 0.0001). The subgroup analysis according to the risk factors is shown in Table 1.

**Table 1:** Incidence of incisional hernia (IH) and average cost per patient in different risk groups.

		Algorithm		
		Yes	No	*P* value
Obesity (*n* = 66)	% IH	6/51(11.8%)	11/15 (73.3%)	<0.001
	COST (€)	10,210 (4,147)	13,588 (6,949)	0.022

≥2 Risk factors (*n* = 97)	% IH	3/61 (4.9%)	16/36 (44.4%)	<0.001
	COST (€)	10,582 (5,088)	10,077 (3,416)	0.561

Low risk (suture) (*n* = 63)	% IH	7/48 (14.6%)	2/15 (13.3%)	1
	COST (€)	9,226 (3,358)	10,279 (4,213)	0.323

Our results suggest that the use of a prophylactic mesh is an effective measure for the prevention of IH in patients undergoing a laparotomy for colorectal resection, at the expense of a significant increase of mild wound complications (seroma). We consider this disadvantage acceptable considering the great benefit it provides in the decrease of IH and the complications derived from this condition and its treatment. Our results are consistent with those previously reported in prospective randomized studies that included all types of laparotomies (3, 5).

The implementation of the algorithm to use prophylactic mesh augmentation in selected patients reduced the incidence of IH and the total hospital costs per patient. Based on our analysis, need for IH repair increases substantially total hospital costs and resource utilization.

In our opinion, not all patients benefit from the routine use of prophylactic mesh augmentation, it should be reserved for patients at increased risk to develop an IH; the rest can be safely closed following the latest recommendations on the published guidelines (6). High-risk cases can be identified with the use of an algorithm, as the one we proposed in our previous publication (19), or different approaches of scoring systems defined by other authors (16, 21).

The use of an algorithm as a therapeutic decision tool has clearly demonstrated its utility in obese patients (BMI > 29 kg/m^2^). In our opinion, there is enough evidence to consider that these patients should receive prophylactic mesh augmentation to prevent IH.

Indeed, if we use the HERNIAscore (HERNIAscore = 4 × L + 3 × HAL + 1 × COPD + 1 × BMI > 25) in our obese patients, we would obtain a minimum of eight points [4 × L + 4 × BMI (29 − 25)], this would be classified as Class III (high risk), resulting in an average risk of IH of 55% (16). When these patients had a prophylactic mesh placed, only 11.8% developed IH, while those who were not treated according to the algorithm presented IH is 73.3% (19).

A significant decrease in the IH rate was also achieved in patients with two or more risk factors associated with IH, other than obesity (1, 14, 15). In this group, we get from 44.4% of IH to 4.9% when the algorithm was applied correctly. In this last group, the algorithm was effective, but there clearly seem to be factors that either have not been considered or have been given an inadequate value. In fact, if we apply the HERNIAscore to these patients, a half of them would have been classified as Class II (moderate risk). This detail indicates that we need to deepen into the analysis of risk factors to elucidate more precisely those patients who benefit from the use of a prophylaxis or the group of patients in whom it is unnecessary, to avoid overtreatment using a mesh in all patients operated on colon and rectum cancer.

In conclusion, we consider that there is enough evidence to recommend the use of selective prophylactic mesh augmentation in high-risk patients, given this measure has proven to be safe and cost-effective in this population.

## Author Contributions

JP has designed the study, written the paper, compiled data, and revised final version. NA has written the paper and compiled data. LH has compiled and analyzed the data and revised the manuscript. ML-C has designed the study and revised the manuscript. MP has designed the study, compiled data, and contributed to discussion and revision of manuscript. JS has revised the manuscript and performed statistical analysis. LG has contributed to discussion and final revision of the manuscript.

## Conflict of Interest Statement

The authors declare that the research was conducted in the absence of any commercial or financial relationships that could be construed as a potential conflict of interest.
